# Ethylene Glycol Poisoning: An Unusual Cause of Altered Mental Status and the Lessons Learned from Management of the Disease in the Acute Setting

**DOI:** 10.1155/2016/9157393

**Published:** 2016-10-25

**Authors:** R. Singh, E. Arain, A. Buth, J. Kado, A. Soubani, N. Imran

**Affiliations:** Detroit Medical Center/Wayne State University, Detroit, MI, USA

## Abstract

Ethylene glycol is found in many household products and is a common toxic ingestion. Acute ingestions present with altered sensorium and an osmolal gap. The true toxicity of ethylene glycol is mediated by its metabolites, which are responsible for the increased anion gap metabolic acidosis, renal tubular damage, and crystalluria seen later in ingestions. Early intervention is key; however, diagnosis is often delayed, especially in elderly patients presenting with altered mental status. There are several laboratory tests which can be exploited for the diagnosis, quantification of ingestion, and monitoring of treatment, including the lactate and osmolal gaps. As methods of direct measurement of ethylene glycol are often not readily available, it is important to have a high degree of suspicion based on these indirect laboratory findings. Mainstay of treatment is bicarbonate, fomepizole or ethanol, and, often, hemodialysis. A validated equation can be used to estimate necessary duration of hemodialysis, and even if direct measurements of ethylene glycol are not available, monitoring for the closure of the anion, lactate, and osmolal gaps can guide treatment. We present the case of an elderly male with altered mental status, acute kidney injury, elevated anion gap metabolic acidosis, and profound lactate and osmolal gaps.

## 1. Introduction

Ethylene glycol is an alcohol found in many household products such as antifreeze, deicing solutions and windshield wiper fluids [1]. Odorless, colorless, and sweet in taste, it is most commonly ingested by children, animals, and those seeking a substitute for ethanol but has also been reported in cases of homicide [2] and suicide. With oral LD_LO_ of 786 mg/kg for humans, which is about 50 milliliters or 1.7 ounces of ethylene glycol for a 70-kilogram patient, even the ingestion of a small volume can prove lethal [3]. The toxicity of ethylene glycol is mediated by its metabolites, mainly glycolic acid and oxalate [4]. These metabolites are responsible for the pathogenesis of the symptoms with which patients present, which are classically those of profound acidosis (altered sensorium and Kussmaul breathing) and crystalluria (consumptive hypocalcemia, oliguria, hematuria, and flank pain) [5].

Diagnosis is made based on known or suspected ingestion in the appropriate clinical setting, with laboratory evaluation playing a key role. While there are tests to specifically identify ethylene glycol in the serum, they may not be readily available and are not without their limitations. As such, clinicians often rely on clinical presentation and findings on blood tests, such as a severe and unexplained anion gap metabolic acidosis with an osmolar gap, to make the diagnosis.

Prevention of sequelae of ethylene glycol intoxication is dependent upon early identification and intervention in order to minimize the production of its toxic metabolites [6]. Early therapeutic intervention is important as most of the ethylene glycol is absorbed through the gastrointestinal tract within one hour of ingestion. The mainstay of treatment is sodium bicarbonate to treat the acute acidosis, fomepizole or ethanol to block conversion of ethylene glycol to its metabolites, and emergent hemodialysis if the ethylene glycol level is more than 50 mg/dL. It should be noted that initial treatment with sodium bicarbonate, though necessary for correction of severe acidosis, may raise the urine pH and increase the likelihood of the precipitation of calcium oxalate crystals, furthering kidney injury and hypocalcemia. This highlights again the importance of early identification and effective intervention. As patients are often critically ill, they frequently require monitoring in an intensive care unit to watch for secondary complications of altered mental status and cardiac arrhythmias. We herein present a case of an accidental ingestion of ethylene glycol in an elderly male that necessitated the above-mentioned therapeutic interventions.

## 2. Case Presentation

A 78-year-old male with a medical history significant for hypertension, dementia, and prior transient ischemic attack was brought into the hospital by his daughter for altered sensorium and gait disturbance. At baseline, the patient was capable of ambulating without assistance. He was last known well around 23:00 one day prior to his emergency visit. According to the daughter, around 11:00 on the day of his admission, the patient was noted to have difficulty in standing and sitting upright unassisted. His speech was garbled and nearly unintelligible. Earlier, he reported to his daughter that he “felt drunk” and was unable to focus on her face. Subsequently, the daughter brought the patient to the emergency room five hours later at around 16:00.

On presentation, the patient was afebrile, normotensive, tachycardic, and tachypneic. Pulse oximetry showed a hemoglobin saturation of 98% on room air. Physical exam findings were normal except for lethargy, disorientation, and response to deep pain by moving all extremities. His blood profile showed an acute kidney injury with creatinine of 1.24 mg/dL, up from a baseline of 1.0 mg/dL (equivalent to drop in glomerular filtration rate from 95 to 68 mL/min/1.73 m^2^), and an elevated anion gap metabolic acidosis with pH of 7.090, PaCO_2_ of 10.0 mmHg, serum bicarbonate of 8 mMol/L, and an anion gap of 29 mMol/L. Lactic acid was 1.6 mMol/L on a venous sample sent to the laboratory; however, a point-of-care arterial blood gas revealed a lactic acid of 27.0 mMol/L (Table 1).

The venous lactic acid level was initially attributed to a laboratory error given the consistently profound low pH on repeated arterial blood gases. A thorough investigation into the cause of this perceived severe lactic acidosis was undertaken and no clear etiology was identified. There was no evidence of sepsis or postictal symptoms, head CT was negative for stroke, and glucose and beta hydroxybutyrate were within normal limits. Furthermore, there were no signs of gangrenous tissue and surgical and radiological evaluations were negative for ischemic bowel or incarcerated hernia. Additionally, the patient was hemodynamically stable with normal liver enzymes, making systemic hypoperfusion or decreased clearance of lactic acid highly unlikely.

Despite several fluid boluses and sodium bicarbonate administration, the acidosis and lactate levels did not improve. Further investigation revealed an osmolar gap of 53 mOsm/kg, an undetectable methanol level, and a serum ethylene glycol level of 54 mg/dL. At this time, repeat lactic acid levels were sent from both venous blood and an arterial blood gas and demonstrated the same significant discrepancy (1.3 versus 22 mMol/L, resp.). Subsequently, the patient was admitted to the intensive care unit and continued on intravenous sodium bicarbonate to correct the underlying acidosis and fomepizole to block conversion of ethylene glycol by alcohol dehydrogenase to toxic metabolites and initiated on emergent hemodialysis. Thiamine and pyridoxine were also given to facilitate conversion of glycolate to less toxic metabolites. Serial labs were drawn to monitor acidosis and ethylene glycol levels. When pH normalized and ethylene glycol levels remained <20 mg/dL, hemodialysis and fomepizole were discontinued.

**Figure 1:**
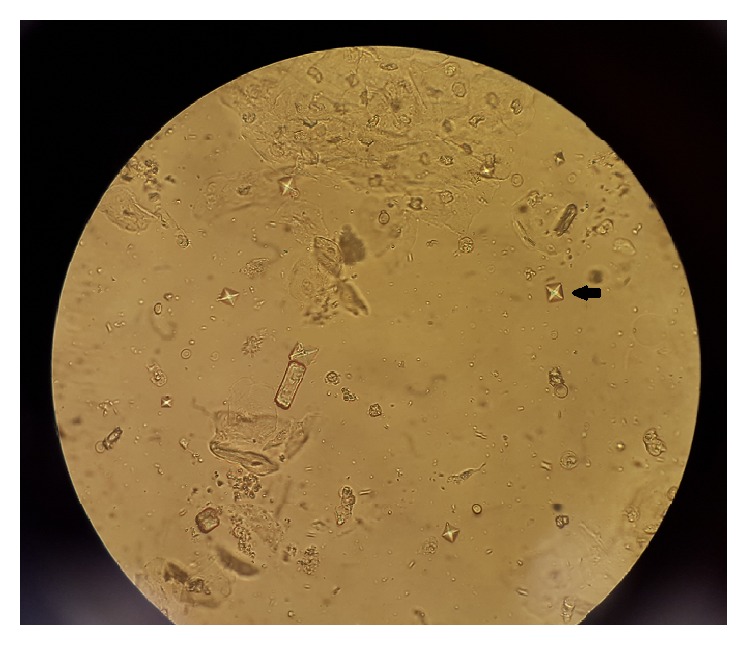
Urine sediment showing envelope-shaped calcium oxalate crystals (arrow).

Initially, renal function was stable, with the patient making nearly 2 liters of urine in 24 hours; however, within 48 hours, the patient became oliguric and his renal function deteriorated. Corrected serum calcium dropped from 9.7 to 7.9 mg/dL, which was attributable to precipitation of calcium oxalate crystals, as examination of urine sediment now revealed the typical envelope-shaped calcium oxalate crystals (Figure 1). Though the patient's mental status improved to his prior baseline, renal function continued to worsen and he became dialysis-dependent. He was discharged on long-term intermittent hemodialysis with uncertain potential for renal recovery.

## 3. Discussion

When patients present to the emergency room with altered mental status, the differential diagnosis is generally broad. While intoxication is always a consideration, ethylene glycol ingestion is often low on the treating physician's radar, especially in a septuagenarian patient. However, ethylene glycol intoxication is not uncommon. In their annual report, the American Association of Poison Control Centers reported a total of 6600 cases of ethylene glycol poisoning during 2013 alone, a steadily rising number over the years. In 2014, this number rose to a total of 6809 cases. In the report, ethylene glycol was the third most common chemical responsible for deaths by nonpharmaceutical poisoning, following ethanol and carbon monoxide [7]. It should be noted though that reporting of these cases to poison control depends solely on the discretion and initiative of the treating physician and, hence, the true number of cases is likely underestimated. In the UK, the National Poisons Information Service reported a total of 91 cases of ethylene glycol poisoning ranking as the fourth most common after acetaminophen, drugs of misuse, and substance abuse. This number only rose to 92 in the following year [8]. This number is significantly smaller than that in the USA but authors will not speculate on the reason and there were no references exploring the differences.

### 3.1. Absorption and Toxicity

**Figure 2:**
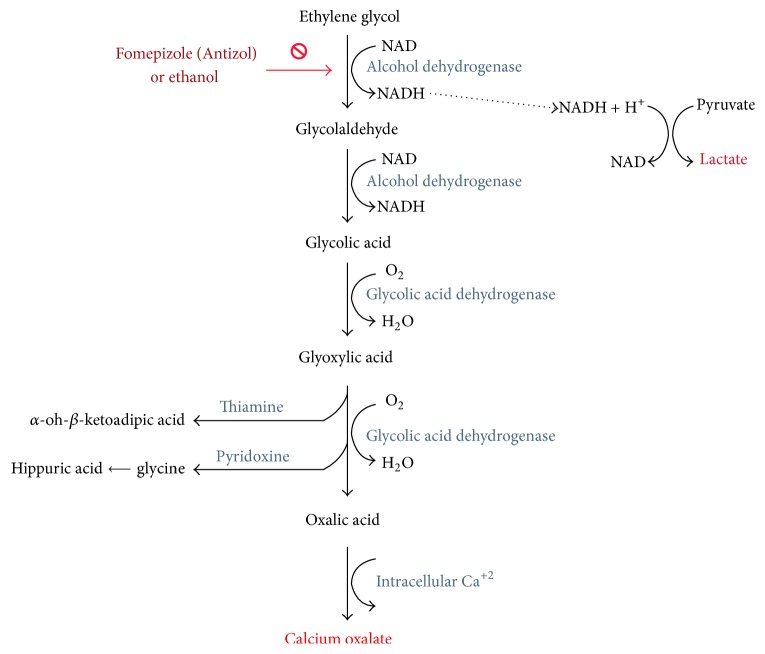
Ethylene glycol metabolism in the human body.

Ethylene glycol is rapidly absorbed in the gastrointestinal tract within the first hour of ingestion. There have been no reported cases of toxicity after dermal or respiratory exposure [9]. Ingestion of ethylene glycol itself results in an inebriated state and central nervous system depression not unlike that seen in ethanol intoxication. The toxicity of ethylene glycol is mediated by its metabolites (Figure 2) [4]. Ethylene glycol, like other alcohols, is metabolized in the liver. Hepatic oxidation via alcohol dehydrogenase and aldehyde dehydrogenase generates glycolaldehyde and then glycolic acid, respectively. Further metabolism leads to glyoxylate, which may be converted to less toxic metabolites if thiamine and pyridoxine are available, and the final end product, oxalic acid.

Circulating glycolic acid in the blood leads to an elevated anion gap metabolic acidosis with resultant Kussmaul breathing and profound obtundation [4]. Direct renal tubular damage was traditionally attributed to glycolic acid, but there is evidence that glycolaldehyde and glyoxylate may be the principle ethylene glycol metabolites responsible for this mechanism of nephrotoxicity and the ensuing acute renal failure, oliguria, and hematuria (Figure 2). This particular damage was found to be independent of the calcium oxalate crystals formation [10]. As oxalate joins with calcium to form calcium oxalate, the resultant hypocalcemia may lead to nerve palsies and tetany. Seizures and arrhythmias may also develop. Calcium oxalate precipitates in urine and leads to further renal injury via crystal induced nephropathy with occasional flank pain [11]. Because ethylene glycol levels peak at one to four hours after oral ingestion and ethylene glycol's half-life is three to nine hours [11], prompt recognition and intervention are imperative to minimize the production of toxic metabolites.

### 3.2. Measuring Ethylene Glycol

Unfortunately, even if a clinician suspects ethylene glycol intoxication, definitive methods of detecting ethylene glycol in the serum have limitations which may significantly delay diagnosis and treatment in a situation where time is of the essence. Gas chromatography is the most common method of detecting serum ethylene glycol, but it is not widely available and is often a “send out” test. In a study by Church and Witting, none of the hospitals surveyed had on-site gas chromatography columns for ethylene glycol testing, and the “send out” turn-around time was greater than one hour [12]. Solvent screens for alcohols may not include ethylene glycol and lead the clinician to incorrectly conclude that no ethylene glycol is present, while enzymatic assays have been known to give false positives in patients with elevated levels of lactate or lactate dehydrogenase [13]. Additionally, patients presenting late may have normal ethylene glycol levels but may be critically ill, having already metabolized ethylene glycol to its toxic metabolites. Therefore, recognition of key findings in more readily available laboratory evaluations in the right clinical setting is of utmost importance. Regarding our patient, quantification of ethylene glycol was performed using the Agilent™ 7890A Gas Chromatography per our center (Detroit Medical Center, DMC) Toxicology University Laboratory protocol. The patient's serum was collected in a red top vacutainer and transported at room temperature. The serum was stored in refrigerator temperatures ranging from 2 to 8°C. Working standards were prepared by mixing the combined standard of 25 mg/dL ethylene glycol and 25 mg/dL propylene glycol with an internal standard containing 1000 mg/dL 1,2-butanediol. BioRad™ drug-free serum and serum volatiles containing ethylene glycol were also used. Controls and patient samples were placed into tubes. A set amount of standard was added to each tube. Controls and patient samples were then transferred to an Amicon™ Filter and the protein-free filtrate was transferred to an injection vial with a micro sample insert. The samples, standards, and controls were run in the Agilent 7890A with Autosampler 6850 per DMC toxicology protocol.

An in-depth evaluation of findings and discrepancies in routine laboratory tests plays a key role in raising clinical suspicion of and diagnosing ethylene glycol intoxication. The profound high anion gap metabolic acidosis seen with ethylene glycol and/or methanol intoxication is seen in few other conditions, and, of those conditions, most also present with severely elevated lactic acid levels, such as bowel ischemia or status epilepticus. Ethylene glycol intoxication may lead to a slight elevation in lactic acid, but it is typically not enough to account for the degree of acidosis [11, 14].

### 3.3. Lactate Gap

One phenomenon which is of great clinical significance and utility is the lactic acid gap. In several studies, it has been noted that lactic acid measurements may be severely but falsely elevated in ethylene glycol intoxication depending on the type of analyzer used. In particular, some point-of-care whole blood analyzers which use L-lactate oxidase may cross-react with glycolate or glyoxylate, leading to massive false-positive lactate elevations [15, 16]. These analyzers are typically used in emergency departments and STAT labs to run arterial blood gases. In contrast, laboratory serum analyzers, which are used for routine analysis of venous blood samples, have less cross-reaction and show very minimal lactate elevation. This lactate gap between an arterial blood gas and venous blood sample is representative then of the presence of toxic ethylene glycol metabolites which are cross-reacting with the point-of-care analyzer. This lactate gap, if present, can be exploited for diagnosis of ethylene glycol intoxication, even in late presentations, as well as for monitoring for clearance of metabolites [16]. In our case, there was a significant lactic acid gap present, which in part prompted investigation into possible ethylene glycol intoxication, and after hemodialysis was initiated, this gap diminished (Table 1).

### 3.4. Osmolal Gap

Alcohol ingestion often also presents with elevated serum osmolal gaps. Although osmolal gaps up to 15 mOsm may be normal based on the method of calculation [17], significantly elevated and unexplained plasma osmolal gaps greater than 20 to 25 mOsm in the right clinical scenario should raise suspicion of the recent ingestion of an alcohol such as ethanol, methanol, ethylene glycol, or isopropyl alcohol. Because the contribution of an alcohol to the osmolal gap is a direct function of that alcohol's molar mass, if the osmolal gap and type of alcohol are known, the amount of that alcohol in the serum can be determined. For example, an osmolal gap of 10 mOsm is equal to 62 mg/dL of ethylene glycol. Our patient had an osmolal gap of 51 mOsm, which suggests an initial level around 316 mg/dL of ethylene glycol. The parent alcohol contributes initially to both the osmolal and anion gaps. However, after ethylene glycol has been metabolized, there will only be an anion gap without the osmolal gap. This phenomenon can help treating physicians trace back to the approximate time of ingestion.

### 3.5. Urine Crystals

Finally, another clue in favor of ethylene glycol ingestion is the appearance of calcium oxalate crystals in the urine and concurrent development of hypocalcemia, presumed to be due to the precipitation of calcium oxalate. However, the finding of crystalluria may not be initially present and may take different forms. The classic dihydrate envelope-shaped crystals are only present at high concentrations of calcium oxalate, with the monohydrate needle-shaped crystals being more common and easily confused with hippurate [11]. It is therefore a late and nonspecific finding.

## 4. Management

Management of ethylene glycol ingestion involves, first and foremost, securing the patient's airway, breathing, and circulation. Severe acidosis and altered sensorium may necessitate endotracheal intubation and hyperventilation. Sodium bicarbonate administration in these patients has two roles, to correct acidosis and to deprotonate glycolic acid and oxalic acid to glycolate and oxalate, making them less likely to penetrate end-organ tissues. However, the delay in hemodialysis initiation to remove oxalate and the continuous bicarbonate infusion may lead to both worsening of hypocalcemia and increasing the risk of calcium oxalate crystal precipitation by raising the urine pH. The next step is to inhibit alcohol dehydrogenase with fomepizole or ethanol. As per current clinical guidelines, fomepizole or ethanol should be given in cases of serum ethylene glycol concentration > 20 mg/dL, known recent ingestion with osmolal gap > 10 mMol, or strong clinical suspicion with three of the following: arterial pH <7.3, serum bicarbonate <20 mMol/L, osmolal gap >10 mMol, or urinary oxalate crystals [18]. Criteria for the initiation of antidotal therapy for known or suspected ethylene glycol intoxication [18] are as follows:

 Serum ethylene glycol of > 20 mg/dL (3.2 mMol/L), or
 Documented recent history of ingestion of toxic amounts of ethylene glycol and an osmolal gap >10 mOsm/L, or
 Suspected ethylene glycol ingestion and at least two of the following:
  1. Arterial pH <7.3
  2. Serum bicarbonate <20 mMol/L (mEq/L)
  3. Osmolal gap >10 mOsm/L
  4. Oxalate crystalluria.

It should be noted that these guidelines are based on expert opinions not on clinical studies. Hemodialysis is indicated if there is severe acidosis, ethylene glycol levels >50 mg/dL, or evidence of end-organ damage, that is, renal failure. Fomepizole is preferred due to easier dosing and decreased side effects [19]. Thiamine, magnesium, and pyridoxine act as cofactors in different reactions that facilitate the conversion of glyoxylic acid to less toxic metabolites such as glycine (and later hippuric acid) and *α*-Hydroxy-*β*-Ketoadipic acid. However, their role is minor and clinicians should not solely rely on them in treating the acute toxicity (Figure 2) [11].

### 4.1. Role of Hemodialysis

As stated above, in patients with severe poisoning, hemodialysis may be life-saving. The consensus is to start hemodialysis if the level of ethylene glycol is more than 50 mg/dL. Having a normal glomerular filtration rate is not a deterrent to initiate hemodialysis. Intravenous fluid should be switched to normal saline to avoid excessive bicarbonate load and to decrease the risk of calcium oxalate crystals precipitation by raising the urine pH. The calcium level will be maintained during the entire hemodialysis session as calcium concentration in the dialysate is at physiological level. If the patient is receiving fomepizole, then it is crucial to increase the dosing frequency from every 12 hours to four hours as fomepizole is significantly dialyzable. Hemodialysis should be continued until the level of ethylene glycol is consistently < 20 mg/dL with successful resolution of metabolic acidosis [5]. The duration of hemodialysis depends on the level of ethylene glycol upon initiation. A validated equation can be used to estimate the time needed in hours: −*V*  [ln⁡(5/*A*)]/[0.06*k*], where *V* is total body water in liters, ln is the natural logarithm, *A* is the initial ethylene glycol concentration in mMol/L, and *k* is 80 percent of the dialyzer urea clearance in mL/min, which is manufacturer-specific and can be easily found using internet search engines [20]. In our patient, weight was 90 kg (45 kg of water), ethylene glycol level upon initiation of dialysis was 54 mg/dL (equal to 8.7 mMol/L), and the dialyzer urea clearance of the filter we used was 266 mL/min at a blood flow of 300 mL/min. We estimated the required duration of hemodialysis to be two hours. Had hemodialysis been started when his presumed ethylene glycol level was 316 mg/dL, he would have required more than 8 hours of hemodialysis.

### 4.2. Predictors of Renal Recovery

There are not many dedicated studies about predictors of renal recovery after ethylene glycol poisoning. In a retrospective study by Lung et al., patients who had lower chances of recovery were more likely to present with coma or seizures, have profound acidosis, or receive therapy (fomepizole or dialysis) more than 6 hours of presentation [21]. It should be noted that most of cases in the literature involve young patients, and so the recovery likelihood may be higher in this population. Unfortunately, our patient was elderly, had other comorbidities, and presented late with a severe acidosis, giving him a lower chance for recovery.

## 5. Conclusion

Diagnosing an elderly and demented patient with ethylene glycol toxicity is very challenging. This patient population can present with metabolic acidosis and confusion due to many etiologies and is at risk of decompensating rapidly. Hence, following the well-established algorithms in assessing patients with metabolic acidosis remains the most effective tool to prevent missing any future cases of ethylene glycol ingestion. Given the vague circumstances around our patient's ingestion incidence, we felt the need to alert adult protective services in order to rule out any malicious intent by the caregiver. The patient is still dialysis-dependent and no recovery is expected soon as he remains anuric.

## Figures and Tables

**Table 1:** Lactic acid and ethylene glycol levels at approximate times in case report.

Measurement	18:00	23:00	02:00	07:00^d^	10:00	12:00^e^
Lactic acid from arterial blood gas, mMol/L	6.4^a^	27^b^	24			3.2
Lactic acid from plasma, mMol/L	1.6^c^			1.3		1.6
Ethylene glycol from plasma, mg/dL			54	30	10	6

^a^Point-of-care ABG run in the emergency department using Radiometer ABL 800 analyzer.

^b^Point-of-care ABG run in the ICU using Radiometer ABL 800 analyzer. All subsequent ABG run with this analyzer.

^c^Laboratory lactic acid measured in venous blood using Siemens Dimension EXL analyzer.

^d^Hemodialysis initiated.

^e^Hemodialysis terminated (note closure of lactate gap).
